# Search for a Grotthuss mechanism through the observation of proton transfer

**DOI:** 10.1038/s42004-023-00878-6

**Published:** 2023-04-22

**Authors:** Ivan Popov, Zhenghao Zhu, Amanda R. Young-Gonzales, Robert L. Sacci, Eugene Mamontov, Catalin Gainaru, Stephen J. Paddison, Alexei P. Sokolov

**Affiliations:** 1 Chemical Sciences Division, Oak Ridge National Laboratory, Oak Ridge, TN USA; 2 Department of Chemistry, University of Tennessee, Knoxville, TN USA; 3 Department of Chemical & Biomolecular Engineering, University of Tennessee, Knoxville, TN USA; 4 Neutron Scattering Division, Oak Ridge National Laboratory, Oak Ridge, TN USA

**Keywords:** Physical chemistry

## Abstract

The transport of protons is critical in a variety of bio- and electro-chemical processes and technologies. The Grotthuss mechanism is considered to be the most efficient proton transport mechanism, generally implying a transfer of protons between ‘chains’ of host molecules via elementary reactions within the hydrogen bonds. Although Grotthuss proposed this concept more than 200 years ago, only indirect experimental evidence of the mechanism has been observed. Here we report the first experimental observation of proton transfer between the molecules in pure and 85% aqueous phosphoric acid. Employing dielectric spectroscopy, quasielastic neutron, and light scattering, and ab initio molecular dynamic simulations we determined that protons move by surprisingly short jumps of only ~0.5–0.7 Å, much smaller than the typical ion jump length in ionic liquids. Our analysis confirms the existence of correlations in these proton jumps. However, these correlations actually reduce the conductivity, in contrast to a desirable enhancement, as is usually assumed by a Grotthuss mechanism. Furthermore, our analysis suggests that the expected Grotthuss-like enhancement of conductivity cannot be realized in bulk liquids where ionic correlations always decrease conductivity.

## Abstract (web summary)

Although the Grotthuss mechanism of proton transfer is generally considered to be the most efficient means of proton transport, the search for experimental evidence of the mechanism has remained elusive and in certain cases confusing. Here, dielectric spectroscopy, quasieleastic neutron and light scattering, along with ab initio molecular dynamics simulations have been utilized to elucidate the mechanism of proton transfer in pure and 85% aqueous phosphoric acid.

## Introduction

It is commonly accepted that there are two major mechanisms of proton transport^1–7^: (1) the vehicular mechanism, where a proton moves via a molecular entity (i.e., a vehicle); and (2) the structural diffusion mechanism^8^, where protons are transferred between different molecules within a hydrogen bond network. The second mechanism provides more efficient proton transport because it does not require the mobility of entire molecules (vehicles). Furthermore, successive proton transfer enables the possibility of a collective mechanism extending over several molecules, originally proposed by Grotthuss to explain observations in the hydrolysis of water^9,10^. According to several prior studies,^9,11–15^ the Grotthuss mechanism is considered to be the most efficient conductivity process because it implies the collective chain-like proton transfer, where the net charge, in principle, can be transported faster than a proton itself. However, the intensive pursuit to experimentally observe this mechanism has led to confusion along with speculation. Many authors assign the observation of a low energy barrier in ionic conductivity as a fingerprint of a Grotthuss-like mechanism^16,17^. However, a low energy barrier is not a sufficient condition, because vehicular transport of charge may also be governed by a low energy barrier for molecular diffusion, e.g., in dilute salt solutions^18^. Although proposed more than 200 years ago, there is still no direct experimental evidence of its existence in bulk systems.

Phosphoric acid (PA) is an excellent and important system for the examination of proton transport in the bulk, due to its highest intrinsic proton conductivity^19–25^. Indeed, nuclear magnetic resonance (NMR) studies of this liquid revealed proton diffusion significantly faster than phosphorus diffusion, clearly indicating the dominance of proton transfer^20^. Structural diffusion is also confirmed by the rather high proton conductivity of PA at temperatures below its glass transition temperature, where molecular (vehicle) motion is frozen while macroscopic proton transport remains active^26^, and by the strong isotope effect upon hydrogen/deuterium substitution^27^. Moreover, extensive computational studies suggested the existence of correlated proton transfer between several PA molecules^25,28,29^ along short chains of ~2–4 molecules^22^. However, the search for direct experimental evidence of structural diffusion as the principle phenomenon responsible for the high conductivity in PA has not been successful. Neutron scattering provides measurement of proton (or hydrogen) dynamics on molecular/atomistic time and length scales. However, several neutron scattering studies were only able to detect processes with proton displacements significantly larger than expected for proton transfer^23,30^. Moreover, recent NMR studies of ^1^H, and ^17^O diffusion^31^ lead authors to the conclusion that structural diffusion dominates conductivity only in pure PA, while H_3_O^+^ diffusion provides a significant contribution to the conductivity in an 85 wt% aqueous PA solution (85 wt% of PA in water that corresponds to 1:1 molar ratio of PA and water; we label this sample as PA85).

To observe and characterize details of proton transport in pure PA and PA85, we performed Broadband Dielectric Spectroscopy (BDS) studies that extend into the sub-THz region, combined with Depolarized Light Scattering and Quasielastic Neutron Scattering (QENS) studies. Furthermore, ab initio molecular dynamics (AIMD) simulations were undertaken to secure insight into the proton transport mechanism. These studies revealed very short proton jump lengths proving the dominating role of the structural diffusion mechanism for the proton transport. Moreover, proton jumps were observed to be correlated. However, these correlations in proton jumps appear to suppress the conductivity, in contrast to the general expectation for a Grotthuss mechanism enhancement in the conductivity.

## Results and discussion

### Broadband dielectric spectroscopy measurements

**Fig. 1:**
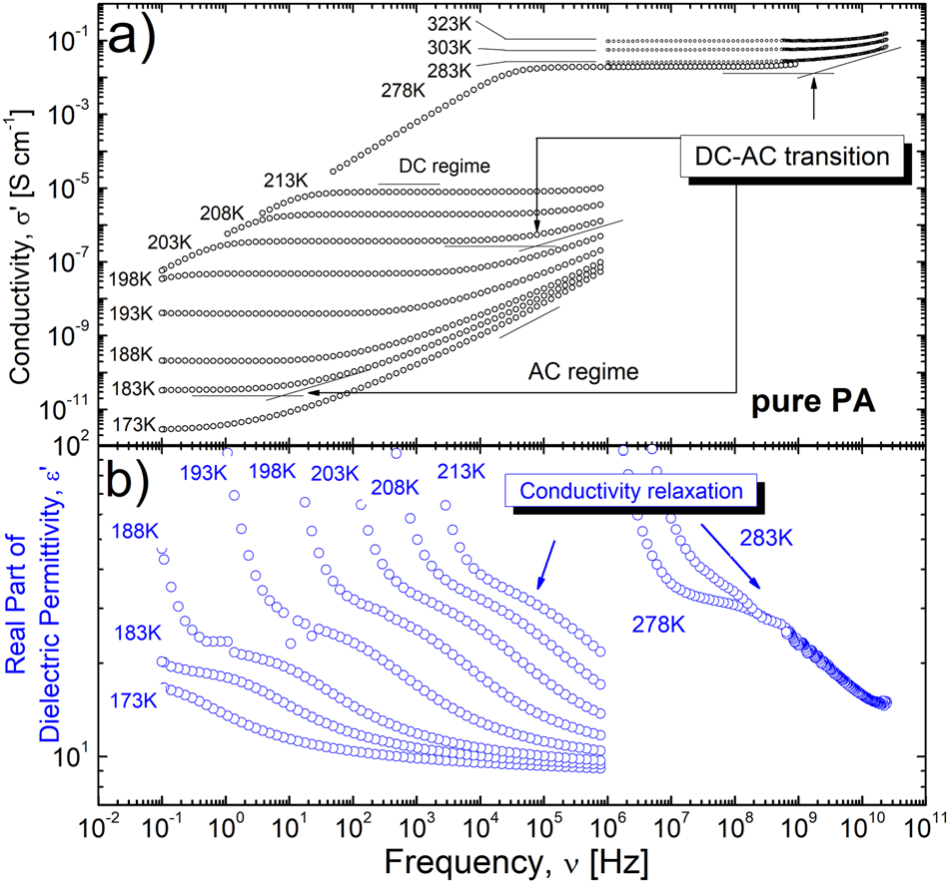
Conductivity and storage permittivity spectra for PA. Conductivity **a** and storage permittivity **b** spectra for PA. Examples of DC-AC transitions **a**, and corresponding conductivity relaxations **b** at different temperatures are marked with arrows. The decrease in conductivity and the increase in storage permittivity at low frequencies are related to electrode polarization.

The conductivity spectra of PA (Fig. 1a; data for PA85 are presented in Supplementary Fig. S1a in Supplementary Information (SI)) show two important regimes: (i) the frequency dependent alternative current (AC) regime at higher frequencies that crosses over to (ii) the frequency independent direct current (DC) conductivity plateau with the amplitude *σ*_DC_ at intermediate frequencies. The AC regime corresponds to the sub-diffusive regime, when the protons are trapped, rattling in Coulombic cages formed by the neighboring counter ions. Once a proton escapes from a cage and begins to diffuse, the conductivity becomes frequency independent and shows a plateau in the DC regime. This escape from a Coulombic cage leads to charge rearrangements that appear as a dipole relaxation also in the real part of the dielectric permittivity (Fig. 1b). Thus, the frequency of the DC-AC transition, *v*_DC-AC_, and its characteristic conductivity relaxation time, *τ*_*σ*_ = 1/(2*πv*_DC-AC_), define the timescale for the transition of charge (i.e., protons) mobility from rattling behavior (in a cage) to a normal diffusion regime^32–38^. The Random Barrier Model (RBM)^32–38^ is often used to describe the conductivity spectra. This model suggests that the transport of charge carriers occurs through hops over potential energy barriers. In the specific case, of a constant barrier height distribution, the model leads to^32,36^:

1 $${{{{\mathrm{ln}}}}}\left(\frac{{\sigma }^{\ast }(\omega )}{{\sigma }_{{{{{{\rm{DC}}}}}}}}\right)=\frac{{{{{{\rm{i}}}}}}\omega {\tau }_{{{{{{\rm{\sigma }}}}}}}{\sigma }_{{{{{{\rm{DC}}}}}}}}{{\sigma }^{\ast }(\omega )}{\left(1+\frac{8}{3}\frac{{{{{{\rm{i}}}}}}\omega {\tau }_{{{{{{\rm{\sigma }}}}}}}{\sigma }_{{{{{{\rm{DC}}}}}}}}{{\sigma }^{\ast }(\omega )}\right)}^{-1/3}.$$

**Fig. 2:**
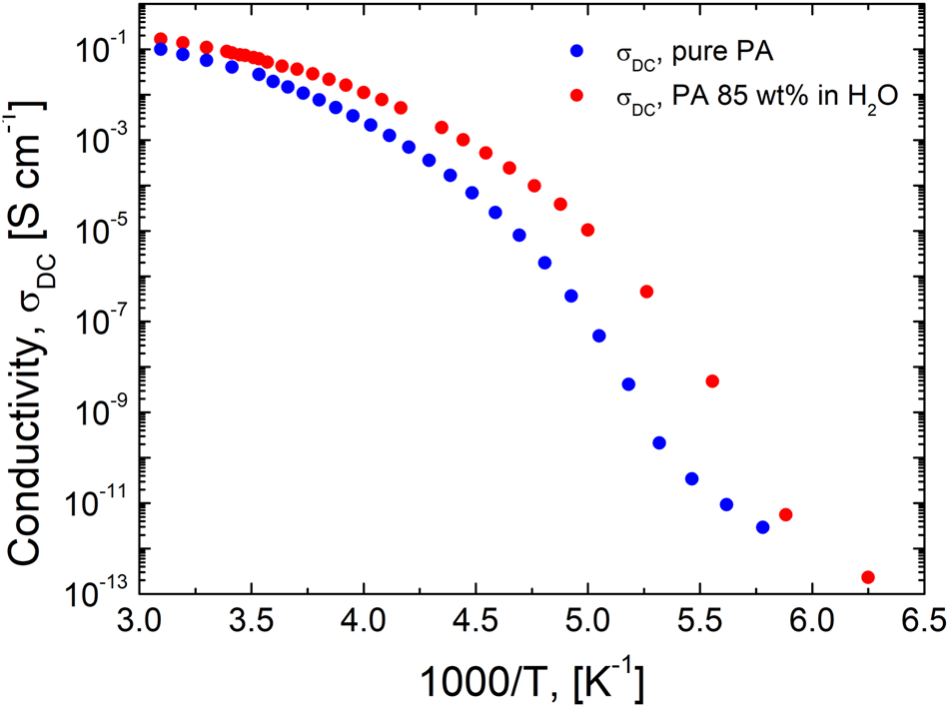
Temperature dependence of the DC-conductivity. Temperature dependence of the DC conductivity, *σ*_DC_, for pure PA (blue symbols) and PA85 (red symbols).

**Fig. 3:**
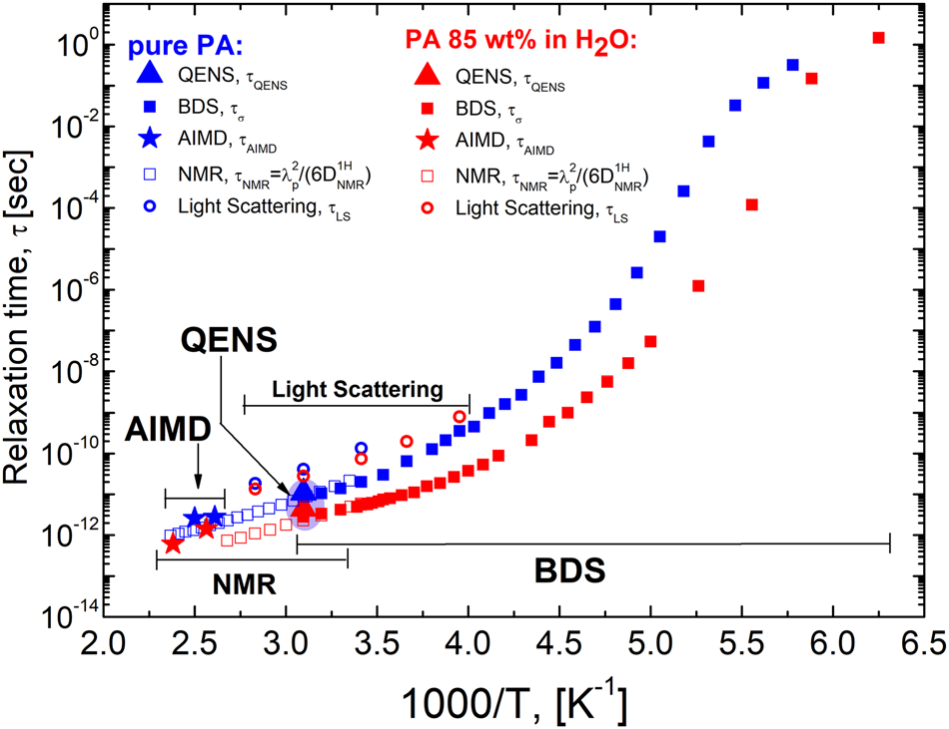
Temperature dependence of the relaxation time. Temperature dependence of the relaxation time obtained with different methods for pure PA (blue symbols) and PA85 (red symbols): Conductivity relaxation time (closed squares); Structural relaxation time from Light Scattering (open circles); Characteristic relaxation time of proton jumps from QENS (triangles) and from AIMD simulations (stars). Characteristic NMR relaxation time for protons (open squares) are estimated from NMR diffusion data, ${\tau }_{{{{{{\rm{NMR}}}}}}}={\langle {\lambda }_{{{{{{\rm{p}}}}}}}\rangle }^{2}/(6{D}_{{{{{{\rm{NMR}}}}}}}^{1{{{{{\rm{H}}}}}}})$, assuming a temperature independent $\langle {\lambda }_{{{{{{\rm{p}}}}}}}\rangle$.

Here σ^*^(ω) is the complex conductivity; *σ*_DC_ is the DC-conductivity, and *τ*_σ_ is the conductivity relaxation time. The RBM also describes the process observed in the permittivity spectra (Fig. 1b). Figure 2 presents the temperature dependence of the DC-conductivity, while Fig. 3 presents the temperature dependence of the conductivity relaxation time estimated from the BDS spectra for pure PA and PA85.

### Light Scattering Measurements

One may wonder whether the relaxation process identified in Fig. 1 may also reflect dipolar relaxation due to the rotation of the PA and water molecules (e.g., as a dielectric *α*-process). In this regard, several experimental observations must be considered: (i) In the highly viscous regime, this conductivity relaxation process is significantly faster than the structural relaxation of the matrix measured by rheology^26,39^; (ii) Despite the strong variation in water content, neat phosphoric acid (Fig. 1b) and its monohydrates (Supplementary Fig. S1b) display similar dielectric relaxation strength; (iii) The dielectric strength of the observed dielectric process decreases upon cooling (Fig. 1b and Supplementary Fig. S1b), this behavior is usual for conductivity relaxation^40–42^ and is opposite to the Curie law (increase in the dielectric strength with cooling) for a dipolar relaxation process^43–46^. All these results suggest that the observed dielectric relaxation process is dominated by conductivity relaxation and is not related to molecular relaxation.

**Fig. 4:**
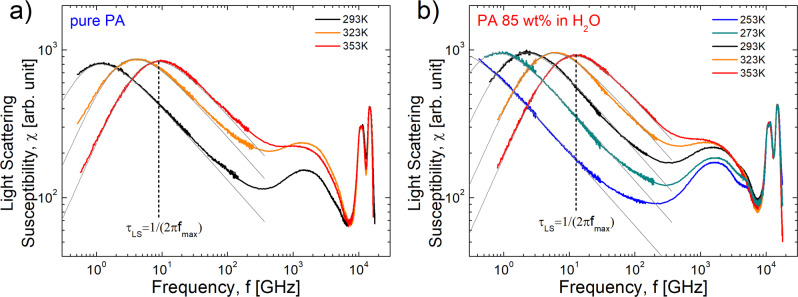
Light scattering susceptibility spectra. Light scattering susceptibility spectra for **a** pure PA and **b** PA85. High frequency Raman modes were used to normalize the spectra at different temperatures. Gray line is the Cole-Davidson fit function^70^. The relaxation time was estimated from the maximum relaxation peak, *τ*_LS_ = 1/2π*f*_max_.

Nevertheless, to estimate the structural relaxation time in these systems, we performed Depolarized Light Scattering (LS) measurements which are not sensitive to the conductivity. The LS susceptibility spectra revealed a relaxation peak (Fig. 4a, b), and the characteristic structural relaxation time can be estimated from the frequency of its maximum, *τ*_LS_ = 1/2π*f*_max_. This estimated structural relaxation time is about one order slower than the conductivity relaxation time (Fig. 3). These results provide clear evidence that the conductivity process in PA and PA85 is significantly faster than the structural relaxation even at high temperatures, and is consistent with the well-known fact that phosphorus in PA moves much slower than protons^20,21^.

Using a proton diffusion coefficient probed by NMR^20,21^ we calculate the characteristic rearrangement length when ‘normal’ diffusion begins with:

2 $$\langle {\lambda }_{{{{{{\rm{p}}}}}}}\rangle =\sqrt{6{D}_{{{{{{\rm{NMR}}}}}}}^{1{{{{{\rm{H}}}}}}}{\tau }_{{{{{{\rm{\sigma }}}}}}}}.$$

The estimates show surprisingly small values $\langle {\lambda }_{{{{{{\rm{p}}}}}}}\rangle$ = 0.7 ± 0.2 Å for pure PA and $\langle {\lambda }_{{{{{{\rm{p}}}}}}}\rangle$ = 0.5 ± 0.2 Å for PA85. This is ~5–6 times smaller than observed in ionic liquids, where $\langle {\lambda }_{{{{{{\rm{ion}}}}}}}\rangle \approx 2.5-3.5{{{{\text{{\AA}}}}}}$ is comparable to the distance between the ions in these systems^37^. However, the estimated $\langle {\lambda }_{{{{{{\rm{p}}}}}}}\rangle$ agrees well with the equilibrium distance ~0.63 Å computed in simulations for proton transfer between PA molecules^25^. These results suggest that PA conductivity is indeed dominated by direct proton transfer between the molecules.

Furthermore, assuming that the rearrangement length is temperature independent, we can use Eq. (2) to determine the conductivity relaxation time of a proton jump at higher temperature based on diffusivity, where the AC-DC crossover in the conductivity spectra is beyond the frequency window. The temperature dependences of ${\tau }_{{{{{{\rm{NMR}}}}}}}={\langle {\lambda }_{{{{{{\rm{p}}}}}}}\rangle }^{2}/(6{D}_{{{{{{\rm{NMR}}}}}}}^{1{{{{{\rm{H}}}}}}})$ and of *τ*_*σ*_(*T*) agree well for both pure PA and PA85 (Fig. 3).

### Quasielastic neutron scattering measurements

**Fig. 5:**
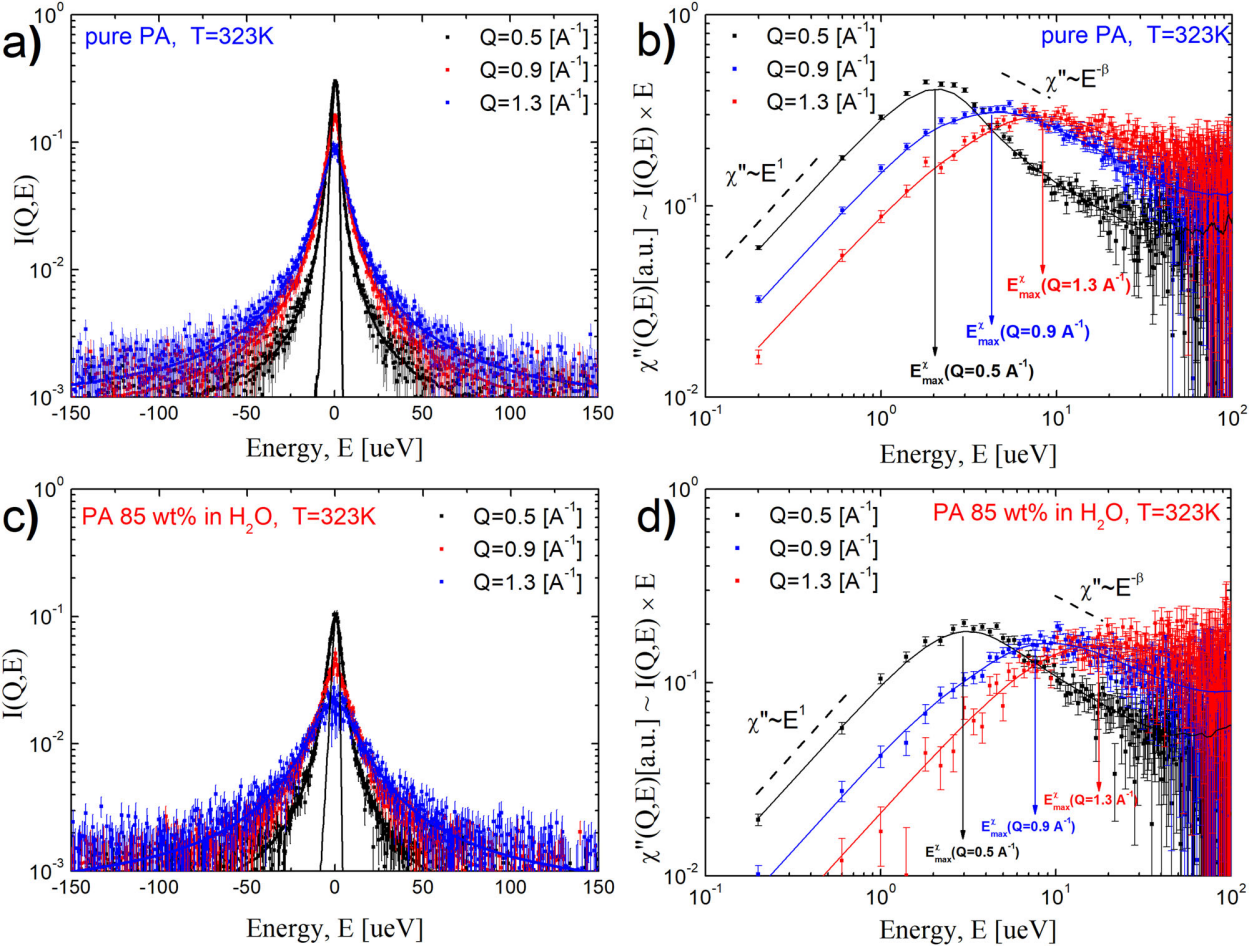
QENS spectra in intensity and susceptibility format. Intensity and corresponding susceptibility presentation of the QENS data, respectively, for pure PA **a**, **b** PA 85 **c**, **d**. Lines present fit to the Cole-Davidson function. For a complete description of the fitting procedure see the SI. The arrows in **b**, **d** show the energies corresponding to the maximum of the relaxation peak in the susceptibility spectra, ${E}_{{{\max }}}^{{{{{{\rm{\chi }}}}}}}(Q)$. This value in the case of the Cole-Davidson function is close to HWHM, but takes into account the distribution of the relaxation processes. In plots **a** and **c** the resolution functions (measured at 20 K) are shown to demonstrate the broadening of the scattering peak. Error bars are standard deviation.

We emphasize, however, that the BDS method does not provide a direct estimate of the proton rearrangement length, only its characteristic rate, and the values of $\langle {\lambda }_{{{{{{\rm{p}}}}}}}\rangle$ are therefore model dependent. In contrast, QENS can provide estimates of the characteristic rearrangement time and length, although the latter is model dependent. Neutrons scatter primarily on hydrogen atoms, and the contribution from oxygen and phosphorus atoms to the neutron scattering spectra of the systems in this study is below 8%. Moreover, oxygen and phosphorus scattering is mostly coherent, and their contribution to incoherent neutron scattering is negligibly small (below 0.1%). Thus, the studied QENS spectra are dominated by motions of protons (hydrogens). The QENS spectra revealed clear broadening that is Q dependent (see Fig. 5a, c), reflecting strong dynamics in the accessible energy range. To analyze the QENS spectra, we convert them to a susceptibility presentation (Fig. 5b, d). The susceptibility spectra reveal a strongly Q-dependent relaxation peak for both PA (Fig. 5b) and PA85 (Fig. 5d). This peak is well described using a Cole-Davidson distribution function with the stretching exponent *β* ≈ 0.68 (for details of the fit procedure see Supplementary Methods in SI). The fit of the spectra (lines in Fig. 5) provides estimates of ${E}_{{{\max }}}^{{{{{{\rm{\chi }}}}}}}(Q)$, which corresponds to the position of the maximum of the relaxation peak in susceptibility format and is close to the Half-Width at the Half-Maximum (HWHM) of the QENS spectra but takes into account the distribution of relaxation processes as well. Analysis also revealed that the remaining elastic component is very small (below 6% for pure PA and below 3% for PA85, see Supplementary Fig. S2a), indicating that all protons in the system, including water protons in PA85, are mobile on the measured time scale. The small remaining elastic component might be related to a scattering from oxygen and phosphorus that are slower than protons.

**Fig. 6:**
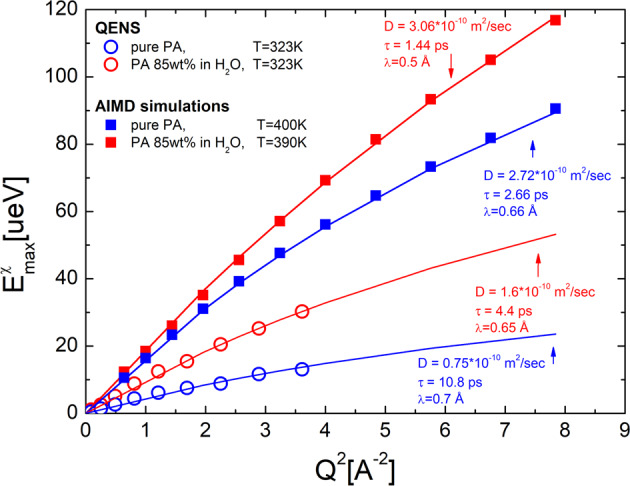
Wave vector dependence of the HWHM. Wave vector dependence of the ${E}_{{{\max }}}^{{{{{{\rm{\chi }}}}}}}(Q)$ for PA (blue symbols) and PA85 (red symbols) estimated from the maximum of relaxation process in the QENS susceptibility spectra at *T* = 323 K (open circles) and from the AIMD simulations at higher *T* (closed squares). The lines show the fit of the AIMD and QENS results to the jump diffusion model (see Eq. (3)).

${E}_{{{\max }}}^{{{{{{\rm{\chi }}}}}}}(Q)$ increases linearly with *Q*^2^ at lower *Q* (Fig. 6), reflecting proton diffusion at long distances, and starts leveling off at higher *Q* corresponding to a finite jump length. This behavior can be well described by the Singwi-Sjolander jump-diffusion model (Fig. 6)^47^:

3 $${E}_{{{\max }}}^{{{{{{\rm{\chi }}}}}}}(Q)=\frac{\hslash {D}_{{{{{{\rm{QENS}}}}}}}{Q}^{2}}{(1+{D}_{{{{{{\rm{QENS}}}}}}}{\tau }_{{{{{{\rm{QENS}}}}}}}{Q}^{2})},{\lambda }_{{{{{{\rm{p}}}}}}}=\sqrt{6{D}_{{{{{{\rm{QENS}}}}}}}{\tau }_{{{{{{\rm{QENS}}}}}}}}$$

Here ħ is the Planck constant divided by 2π, and *τ*_QENS_ and *D*_QENS_ are the characteristic relaxation time and diffusion coefficient of protons. The fit of ${E}_{{{\max }}}^{{{{{{\rm{\chi }}}}}}}(Q)$ to the jump-diffusion model (Fig. 6) results in a diffusion coefficient in good agreement with the NMR data (see Supplementary Fig. S3), confirming that QENS measures proton diffusion at lower *Q*. The most impressive aspect of this is that the estimated relaxation time, *τ*_QENS_, is similar to the value of *τ*_σ_ estimated from the BDS spectra and is much faster than the structural relaxation estimated from the LS spectra in these systems (Fig. 3). The agreement of *τ*_QENS_ and *τ*_σ_ reveals that proton dynamics probed by QENS governs the conductivity of both PA and PA85. QENS also provides model dependent estimates of the jump length (Eq. 3), with $\langle {\lambda }_{{{{{{\rm{p}}}}}}}\rangle$ = 0.7 ± 0.2 Å for pure PA and $\langle {\lambda }_{{{{{{\rm{p}}}}}}}\rangle$ = 0.6 ± 0.2 Å for PA85, in excellent agreement with the estimates from combined BDS and NMR data.

Our results also explain why earlier neutron scattering studies of PA^23,30^ could not detect the proton transfer process. In these previous studies, QENS spectra were measured in a much narrower energy transfer range (±15 or ±25 μeV) and analyzed using a single Lorentzian peak plus background, while HWHM obviously exceeds 20 ‒ 40 μeV at higher *Q* (Fig. 5). As a result, the major part of the faster proton transfer events was missed. Our data clearly show that the proton transfer process is stretched (see Supplementary Methods in SI) and occurs with a characteristic energy *E* ~ ħ/ *τ*_QENS_ ~ 130 μeV.

### Ab Initio Molecular Dynamics Simulations

**Fig. 7:**
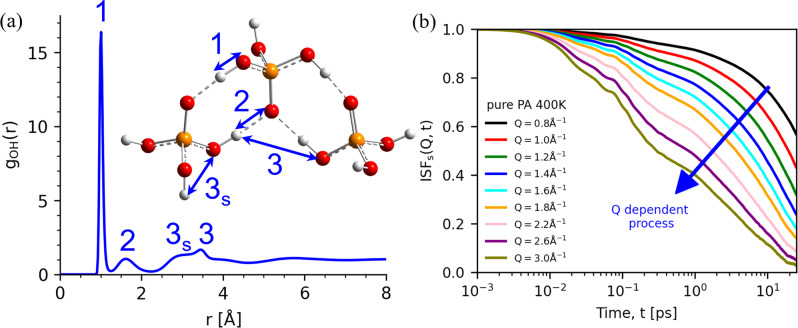
The radial distribution function and self-intermediate scattering function. **a** The radial distribution function (*g*_OH_) of oxygen-hydrogen in PA obtained from AIMD. The shoulder on the left side of the 3^rd^ peak marked 3 s corresponds to the distance between the proton and the oxygen atoms in the same PA molecule. **b** Self-intermediate scattering function *ISF(Q*, *t)* of protons with different *Q* values at 400 K for pure PA.

To unravel the molecular picture of the proton transport in PA we performed AIMD simulations (see Supplementary Methods in SI for a complete description). They allow one to calculate the Self-intermediate Scattering Function of the protons, *ISF*(*Q,t*), which provides similar information to that obtained from QENS data, but can access a much higher *Q* range. The *ISF(Q,t)* shows three processes (Fig. 7b, Supplementary Figs. S4a, S5, and Supplementary Tables S1-S6). The fastest process with *Q*-independent characteristic time occurs at a few femtoseconds. Examination of the vibrational density of state for protons in pure PA indicates that this process is due to the covalent vibration of the hydrogen atoms (Supplementary Fig. S4b). The intermediate process has a weak *Q*-independent time constant ~0.1−0.3 ps, suggesting a local process. Similar processes with characteristic times of ~0.1−0.3 ps were reported in simulations of water and imidazole and assigned to proton rattling between two oxygen/nitrogen atoms^48,49^. Following this idea, we also ascribe this process to the rattling of protons between oxygen atoms in PA and PA85. Furthermore, the proton transfer population correlation function (PCF) validates that the proton rattling process takes place at the sub-picosecond time scale (Supplementary Fig. S6a). The slow process, however, exhibits strong Q dependent relaxation time, *τ*_3_, indicating its diffusive nature. It can be well described by a stretched exponential decay, $\propto exp [-{(t/{\tau }_{3})}^{\beta }]$, with the stretching parameter *β* ~ 0.7 ± 0.2. The latter agrees with the stretching determined from the QENS spectra. Moreover, the HWHM calculated from the obtained *τ*_3_(*Q*), ${E}_{{{\max }}}^{{{{{{\rm{\chi }}}}}}}(Q)=\hslash \beta /[{\tau }_{3}\Gamma (1/\beta )]$, exhibits the same *Q* dependence as the process observed in QENS (Fig. 6). The fit of the AIMD simulation results (Fig. 6) provides estimates of the parameter $\langle {\lambda }_{{{{{{\rm{p}}}}}}}\rangle$ = 0.7 ± 0.2 Å for pure PA and $\langle {\lambda }_{{{{{{\rm{p}}}}}}}\rangle$ = 0.5 ± 0.1 Å for PA85, in excellent agreement with the QENS data analysis. The same fit provides the value of relaxation time and is plotted in the Fig. 3 in good agreement with our experimental results.

**Fig. 8:**
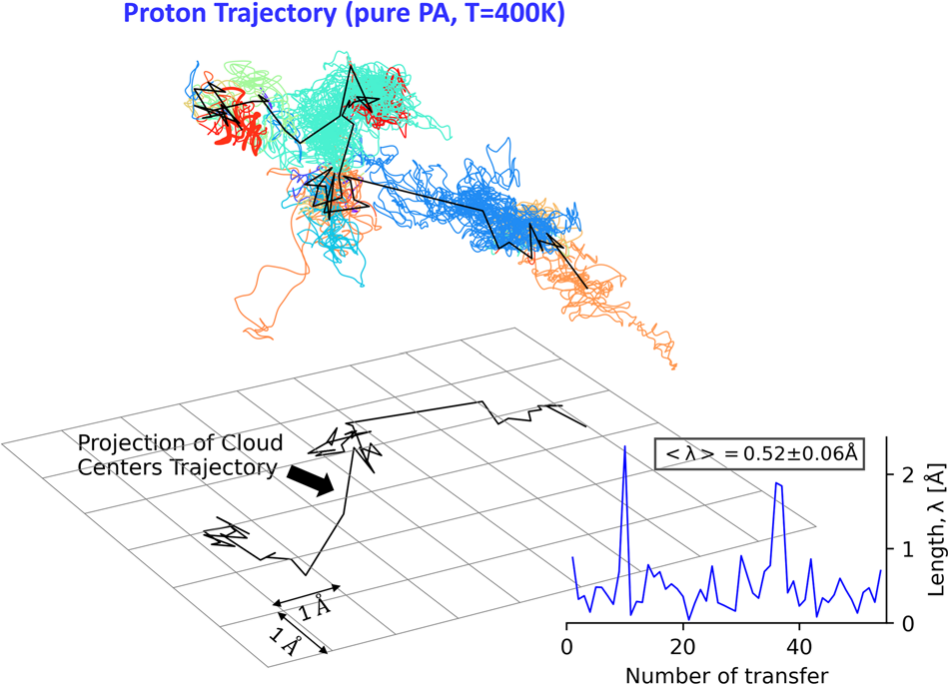
Trajectory of an individual proton in pure PA. Trajectory lines of an individual proton in pure PA at 400 K. Each colored cloud reflects the proton vibrational and rotational motions as the proton is associated with the same oxygen atom. The trajectory changes color when the proton transfers to a different oxygen atom. The center of mass of each cloud is connected by a black line to the center of mass of the next cloud. These black lines present averaged jumps of the proton transfers between the oxygen atoms. (Inset) The length of each proton jump between oxygen atoms (the length of the black lines). The average jump length is very short ~ 0.5 Å, although there are a few larger (~2 Å) jumps that are associated with the large-angle jumps followed by a proton transfer.

Having established a clear relationship of the diffusive process in the simulations to the proton jumps observed in the QENS, we can now follow the trajectory of an individual proton during its diffusion over 40 ps (Fig. 8). Trajectories show the vibrational and rotational motion of the proton within the same color cloud and proton jumps are defined as the proton transfers from one oxygen atom to another oxygen atom (black lines). Analysis of these proton jumps reveals that they are very short ~ 0.5 Å with only a few (~2–3 out of 50) large scale jumps ~ 2 Å. This is smaller than that suggested much earlier jumps (~2.8 Å)^20^. Specifically, the short proton jumps are the proton transfers between the same pair of oxygen atoms although there are some trials of rotating motions. However, the long jumps are associated with a rotation of the OH bond followed by an effective proton transfer, which is a much rarer event than individual proton jumps. Examination of the other protons in the PA system shows a similar distribution and average jump length.

**Fig. 9:**
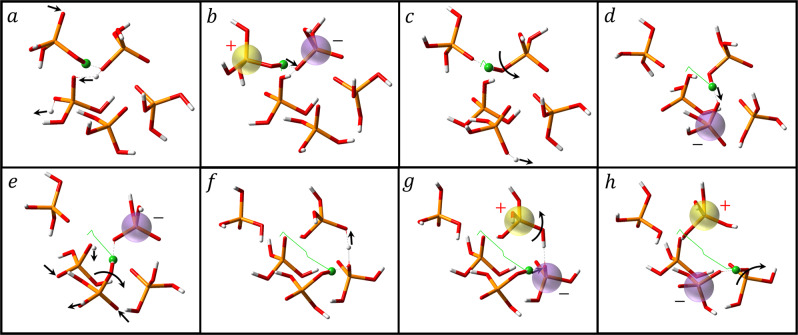
Snapshots of proton transport. The length of each proton jump between oxygen atoms for a specific proton. The average jump length is very short ~0.5 Å. **a**–**h** Snapshots of the elementary steps of the mechanism for long-range proton diffusion in pure PA. The charged entities are highlighted. The collaboration of proton transfers **a**, **b**, **d**, **f**, **g** and large angular jumps **c**, **e**, **h** facilitates the long-range diffusion of protons. See the text for a detailed description.

The AIMD simulations reveal that proton hopping plays a key role in the long-range proton diffusion through the breaking and forming of hydrogen bonds (Fig. 9). The structural characteristics of a proton with neighboring oxygen atoms show that the covalent OH bond and the hydrogen bond are the first peak at *r*_OH_ = 1.01 Å and the second peak at *r*_OH_ = 1.61 Å, respectively, in the radial distribution function g_OH_ (Fig. 7a). The proton (in green) first forms a strong hydrogen bond with a neighboring oxygen, which induces the proton associated with the neighboring oxygen to transfer to the solvent (neutral) PA molecule (Fig. 9a). Following the completion of the proton transfer from the neighboring oxygen to the solvent PA molecule, the proton transfers to the neighboring oxygen through a strong hydrogen bond (Fig. 9b). These successive proton hopping events take place at the sub-picosecond time scale, followed by a long quiescent period, in which the proton is trapped in a cage formed by adjacent oxygen atoms via strong hydrogen bonds. This quiescent period corresponds to the hydrogen bond reorganization relaxation time (a couple of picoseconds), which is also revealed by the proton transfer PCFs (Supplementary Fig. S6a). The large-angle proton jump due to the reorientation of the covalent OH bond causes the breaking of the original hydrogen bond and the forming of the nascent hydrogen bond with a new PA molecule (Fig. 9c, d), which facilitates protons escaping the local “trap” formed by the strong hydrogen bonds. Subsequently, proton transfer occurs via the new hydrogen bond followed by another large-angle reorientation of the covalent OH bond (Fig. 9e, f). The proton transfer through this newly formed hydrogen bond induces the cleavage of the other hydrogen bond between the next acceptor oxygen and the surrounding solvent PA molecules by a large reorientation of a covalent OH bond (Fig. 9g, h). This molecular mechanism indicates that the reorientation of an OH bond by large angular jumps is involved in the long-range diffusion of the protons.

### Proton correlations

Once the molecular-level picture of the proton jumps and its averaged length are established, we can finally analyze the proton dynamics correlations and the existence of a Grotthuss-like mechanism in the conductivity. Historically, ion correlations in dilute solutions were described by Debye-Huckel-Onsager theory^50^. However, this approach is valid only for dilute solutions^50^. Traditionally, correlations in concentrated ionic systems are analyzed from comparison of the experimentally measured conductivity, *σ*_DC_, to that expected for uncorrelated ion diffusion, i.e., conductivity estimated using the Nernst-Einstein (NE) equation, *σ*_NE_^51,52^:

4 $${H}^{-1}=\frac{{\sigma }_{{{{{{\rm{DC}}}}}}}}{{\sigma }_{{{{{{\rm{NE}}}}}}}},\,{{{{{\rm{where}}}}}}\,{\sigma }_{{{{{{\rm{NE}}}}}}}=\frac{n{q}^{2}D}{{k}_{{{{{{\rm{B}}}}}}}T}.$$

Here *n*, *q*, and *D* are the concentration, charge, and diffusion coefficient of the mobile ions, respectively. The parameter *H*^−1^ is the inverse Haven ratio, often called ‘ionicity’, characterizes the ion transport correlations^53,54^, and can be used as an indicator of the presence of a Grotthuss-like mechanism. Indeed, measured DC conductivity by definition depends on velocity-velocity correlations of all the ions^51,55,56^

5 $${\sigma }_{{{{{{\rm{DC}}}}}}} =\frac{1}{3V{k}_{{{{{{\rm{B}}}}}}}T}\int_{0}^{\infty }\left\langle {\overrightarrow{{{{{{\bf{J}}}}}}}(0)}\cdot \overrightarrow{{{{{{\bf{J}}}}}}}(t)\right\rangle {{{{{\rm{d}}}}}}t,\,{{{{{\rm{where}}}}}}\,\\ \left\langle \overrightarrow{{{{{{\bf{J}}}}}}}(0)\cdot \overrightarrow{{{{{{\bf{J}}}}}}}(t)\right\rangle =\left\langle {\sum }_{i}{q}_{{{{{{\rm{i}}}}}}}{\overrightarrow{{{{{{\bf{v}}}}}}}}_{{{{{{\rm{i}}}}}}}(0)\cdot {\sum }_{{{{{{\rm{j}}}}}}}{q}_{{{{{{\rm{j}}}}}}}{\overrightarrow{{{{{{\bf{v}}}}}}}}_{{{{{{\rm{j}}}}}}}(t)\right\rangle.$$

**Fig. 10:**
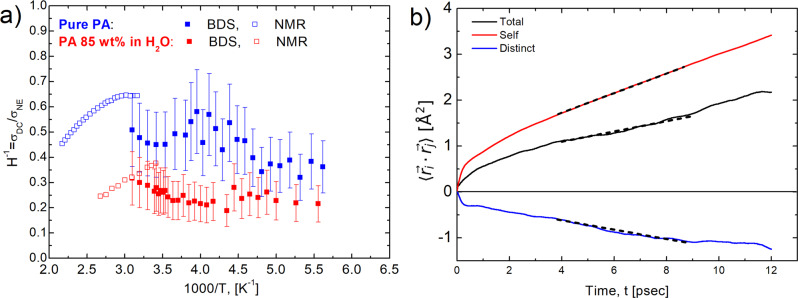
Inverse Haven ratio and correlation functions of proton displacement vectors. **a** Temperature dependence of the inverse Haven ratio for PA (blue symbols) and PA85 (red symbols) estimated using diffusion coefficients obtained from NMR^20,21^ (open squares) and BDS (closed squares). Error bars are standard deviation. **b** Correlation functions of proton displacement vectors for pure PA at 400 K and the linear fit to obtain self-, distinct, and total diffusion coefficients using Eq. (6).

Thus, DC conductivity can be presented as *σ*_DC_ = *σ*_NE_ + *σ*_++_ + *σ*_+−_ + *σ*_− −_, where the last three terms show contributions of distinct ion-ion correlations^57–59^. As a result, the inverse Haven ratio can be expressed as ${H}^{-1}=1+{\sigma }_{++}/{\sigma }_{{{{{{\rm{NE}}}}}}}+{\sigma }_{+-}/{\sigma }_{{{{{{\rm{NE}}}}}}}+{\sigma }_{--}/{\sigma }_{{{{{{\rm{NE}}}}}}}$. The original idea of the Grotthuss-like mechanism implies that protons move in the same direction (chain-like)^12^, enhancing conductivity and leading to *σ*_++_ > 0. Furthermore, in PA and PA85 we can neglect the contribution of *σ*_+−_ and *σ*_−−_ in comparison to *σ*_++_ because of slow anion diffusion in comparison to proton diffusion. Thus, if Grotthuss-like mechanism takes place in PA or PA85, the inverse Haven ratio has to be larger than one, *H*^−1^ > 1^12,19,60^. The low value of the elastic component in QENS spectra (within the error bars) indicates that all protons are mobile and participate in conductivity, even the water protons in PA85 (Supplementary Fig. S2a). The DC conductivity *σ*_DC_ is directly extracted from the dielectric spectra and it is plotted in Fig. 2 as a function of temperature. To estimate *σ*_NE_, we used proton diffusivities from NMR at higher temperatures^20,21^, and estimations based on the relaxation time from BDS data at lower temperatures, assuming temperature independent $\langle {\lambda }_{{{{{{\rm{p}}}}}}}\rangle$, $D={\langle {\lambda }_{{{{{{\rm{p}}}}}}}\rangle }^{2}/(6{\tau }_{{{{{{\rm{\sigma }}}}}}})$. Analysis reveals that *H*^−1^ < 1 in the entire temperature range (Fig. 10a). The obtained estimates of the inverse Haven ratio agrees with many earlier studies^19,20^, and indicates that proton transfer between PA molecules is indeed correlated. However, in contrast to the expected enhancement in the conductivity due to a Grotthuss-like mechanism, the correlated proton transfer in PA leads to a decrease in conductivity (Fig. 10a).

To confirm this result, we also analyzed distinct proton-proton displacement vector correlations from the AIMD simulation trajectories. The self- and distinct- diffusivities of the protons may be obtained using the Einstein relation:

6 $${D}_{ij}=\frac{1}{6}\mathop{{{{{\mathrm{lim}}}}}}\limits_{t\to \infty }\frac{{{{{{\rm{d}}}}}}}{{{{{{\rm{d}}}}}}t}\left\langle \mathop{\sum }\limits_{i=1}^{N}\mathop{\sum }\limits_{j=1}^{N}[\overrightarrow{{{{{{{\bf{r}}}}}}}_{i}}({{{{{\rm{t}}}}}})-\overrightarrow{{{{{{{\bf{r}}}}}}}_{i}}(0)]\cdot [\overrightarrow{{{{{{{\bf{r}}}}}}}_{j}}({{{{{\rm{t}}}}}})-\overrightarrow{{{{{{{\bf{r}}}}}}}_{j}}(0)]\right\rangle ,$$

where $\overrightarrow{{{{{{{\bf{r}}}}}}}_{i}}$ and $\overrightarrow{{{{{{{\bf{r}}}}}}}_{J}}$ are the coordinates of protons *i* and *j*, respectively. Equation (6) can be decomposed into the self- (${D}_{ii}^{{{{{{\rm{self}}}}}}}$ for *i* = *j*) and distinct- (${D}_{ii}^{{{{{{\rm{distinct}}}}}}}$ for *i* ≠ *j*) diffusivity contributions. Assuming that the proton conductivity is the dominant contribution to the conductivity of the system, the inverse Haven ratio can be computed via ${H}^{-1}=({D}_{ii}^{{{{{{\rm{self}}}}}}}+{D}_{ii}^{{{{{{\rm{distinct}}}}}}})/{D}_{ii}^{{{{{{\rm{self}}}}}}}$. These diffusivities were calculated from the linear fitting to the proton displacement vector correlations $(\langle \overrightarrow{{{{{{{\bf{r}}}}}}}_{i}}\cdot \overrightarrow{{{{{{{\bf{r}}}}}}}_{j}}\rangle )$. Indeed, the distinct proton correlation is negative, ${D}_{ii}^{{{{{{\rm{distinct}}}}}}}=-1.53\times {10}^{-6}{{{{{{\rm{cm}}}}}}}^{2}{{{{{{\rm{s}}}}}}}^{-1}$, leading to a negative contribution to the conductivity (Fig. 10b). Given that the negative distinct correlation of a proton may arise through the rotation of PA molecules, we calculated the distinct correlation function of the proton displacement vectors from the same PA molecule and the results show that the distinct diffusivity of protons from the same PA molecule are very small with a positive value (Supplementary Fig. S7). Notably, the self-diffusion coefficient of the protons is in good agreement with that obtained from the intermediate scattering function $({D}_{ii}^{{{{{{\rm{self}}}}}}}=3.59\times {10}^{-6}{{{{{{\rm{cm}}}}}}}^{2}{{{{{{\rm{s}}}}}}}^{-1})$. The anticorrelation between protons definitely gives rise to the suppression of the conductivity in pure PA at 400 K (*H*^−1^ = 0.58). This combination of experimental and simulations results suggests that correlated proton transfer events in PA and PA85 form a kind of backflow with total charge displacement lower than in the case of uncorrelated proton transfer. The case where *H*^−1^ < 1 is typically observed in ionic liquids^61,62^ and is explained by momentum conservation between the moving ions. Thus, we suggest that a Grotthuss chain-like proton conductivity is impossible in a bulk liquid state. It usually leads to a suppression of conductivity by anion-anion and cation-cation correlations^57,58^.

## Conclusion

In summary, our results demonstrate that the proton jump length is much shorter than the distance between the hydrogen atoms in the system and is comparable to the distance of proton transfer between oxygen atoms forming a hydrogen bond either between two PA molecules, or PA and water molecules in the case of PA85. The strong similarity in the results for PA and PA 85 suggests that proton transfer is also the dominating conductivity mechanism in PA85. It is known that adding water to PA reduces viscosity and increases conductivity^26^. This indicates that water molecules assist in faster proton diffusion with respect to neat PA. However, also for PA85 this diffusion involves short proton jumps between molecules rather than vehicular diffusion (i.e., as H_3_O+). To the best of our knowledge, the presented combination of several techniques provides the first experimental observation of proton jumps between molecules as the dominant proton diffusion mechanism in a bulk system.

## Methods

### Materials

Phosphoric acid (PA) (crystalline, ≥99.99% trace metal basis) was purchased from Sigma-Aldrich and 85 wt% phosphoric acid aqueous solution (i.e., 85% of the weight of PA in water) was purchased from Alfa Aesar. Both were used as purchased. The sample PA 85 wt% in H_2_O was chosen for more detailed measurements due to its stability at ambient temperature and lower sensitivity to moisture. For BDS, QENS, and Light Scattering measurements pure PA samples were loaded into the cell in a glovebox under dry argon atmosphere. Pure PA, below crystallization temperature was measured only in a liquid supercooled state.

### Broadband Dielectric Spectroscopy (BDS) measurements

Spectra of complex conductivity measurements were probed using three setups to cover the wide frequency range from 0.1 Hz up to 30 GHz. An Alpha-A analyzer from Novocontrol was utilized in the frequency range of 0.1 Hz to 10^6^ Hz. An Agilent RF Impedance Material Analyzer, E4991A was used in the frequency range 10^6^ Hz to 3 × 10^9^ Hz. The Panoramic Network Analyzer, Agilent Technologies, E8364C with 85070E Dielectric Probe Kit were utilized for frequency measurements from 5 × 10^8^ Hz up to 3 × 10^10^ Hz. The Slim Probe with the Agilent Electronic Calibration module (ECal) were used for measurements of real and imaginary part of dielectric permittivity. A Quattro temperature controller (Novocontrol) was used for temperature stabilization for the measurements from 10^−1^ Hz to 10^9^ Hz. The samples were stabilized for 20 min at each temperature to reach precision ±0.2 K. The Presto, Julabo, W80 was used for temperature stabilization for the measurements from 5 × 10^8^ Hz up to 3 × 10^10^ Hz. The samples were stabilized for 40 min at each temperature to reach precision ±0.2 K. Additional details of calibration and designs of the cells are presented in Supplementary Methods in SI.

### Quasielastic neutron scattering (QENS) measurements

at the BASIS spectrometer (Spallation Neutron Source, ORNL)^63^ were performed in the extended energy transfer mode to cover the energy transfer window of −200 µeV to +200 µeV at 323 K for pure PA and PA 85 wt% aqueous solution. This was achieved with the spectrometer’s bandwidth choppers operated at 30 Hz and the incident neutron bandwidth centered at 6.15 Å. The energy resolution was ca. 3.7 µeV (Q-averaged full width at half maximum). The samples were placed in flat-plate gold-coated aluminum sample holders with sample space thickness of 0.25 mm to reduce multiple scattering. Samples were loaded in glovebox and sealed with indium. The sample holder was placed at 90° with respect to the incident beam. A closed-cycle refrigerator was used for temperature control within 0.5 K offset. The sample-specific resolution spectra were collected at a baseline temperature of 20 K. The QENS data were reduced and analyzed using Mantid^64^ and DAVE^65^ software, respectively. Additional details of data treatment are presented in Supplementary Methods in SI.

### Light scattering (LS)

was measured using Raman spectrometer and Tandem Fabry-Perot (TFP - 1) interferometer (JRS Scientific Instruments, Table Stable Ltd). The experiments were performed in backscattering geometry using laser wavelength 532 nm. To obtain wide frequency range, three mirror spacing were used in TFP interferometer: 0.4 mm, 3 mm, and 15 mm. Narrow interference filter was used to suppress higher order transmissions. Optical signal from the same spot in the sample was transferred by additional mirrors to the T64000 Raman spectrometer from Horiba Jobin Yvon. Raman spectra were measured in subtractive mode, and the optical Raman modes were used to normalize the spectra at different temperatures. A glovebox was used to load the samples in a glass vial and seal. The samples were measured in cryostat. After temperature stabilization for one hour, the light scattering spectra were collected. To remove the contribution of longitudinal modes from the light scattering spectra, the samples were measured in HV and HH polarizations at the same mirror spacing.

### Ab initio molecular dynamics (AIMD) simulations

AIMD simulations of pure PA and 85 wt% aqueous PA were performed using the CPMD code^66^. The pure PA system was made up of 54 PA molecules in a cubic simulation box of side *L* = 16.696 Å (*ρ* = 1.885 g cm^−3^), and the PA85 system consisted of 38 PA molecules and 38 H_2_O molecules in a cubic simulation box of side *L* = 16.317 Å (*ρ* = 1.685 g cm^−3^). The dispersion-corrected atomic core pseudopotentials (DCACP)^67,68^ were employed to account for dispersion forces within the Kohn-Sham formulation of density functional theory and the B-LYP exchange-correlation functional^69^. A plane-wave basis set was used to expand the Kohn-Sham orbitals up to a kinetic energy cutoff of 80 Ry. In order to increase the adiabatic decoupling of nuclear and electronic dynamics, and allow a larger time step 4 au, the mass of deuterium was assigned to the hydrogen atoms and the fictitious electron mass was set to *μ* = 500 au. Each system was pre-equilibrated using a classical force field for 10 ns, followed by 15–20 ps equilibration with AIMD at the desired temperatures using a massive Nose-Hoover chain thermostat. Note that the temperature of the simulated system was increased to enhance proton dynamics without changing the physical mechanism and to avoid the formation of a glassy state because the expansive AIMD simulations limit the temporal and spatial scales. Subsequently, the equilibrated configuration was used for the ~60 ps production run in a microcanonical (NVE) ensemble.

## Supplementary information

Supplementary Information

## Data Availability

The data that support the plots within this paper and another finding of this study are available from the corresponding author upon reasonable request.

## Code availability

The computer code used for simulations is available from the corresponding authors upon reasonable request.
