# Supplementary material for: Search for a Grotthuss mechanism through the observation of proton transfer
Source: Commun Chem. 2023 Apr 22;6:77. doi: 10.1038/s42004-023-00878-6 (PMC10122652; doi:10.1038/s42004-023-00878-6)
Supplement: Supplementary file 1 — Supplementary Information [file 42004_2023_878_MOESM1_ESM.pdf]

Supplementary Information

Search for a Grotthuss mechanism through the observation of proton transfer

Ivan Popov[1,2], Zhenghao Zhu[3], Amanda R. Young-Gonzales[2], Robert L. Sacci[1], Eugene Mamontov[4], Catalin Gainaru[1], Stephen J. Paddison[3*], Alexei P. Sokolov[1,2**]

[1]Chemical Sciences Division, Oak Ridge National Laboratory, Oak Ridge, Tennessee 37831, USA

[2]Department of Chemistry, University of Tennessee, Knoxville, Tennessee 37996, USA

[3]Department of Chemical & Biomolecular Engineering, University of Tennessee, Knoxville, Tennessee 37996, USA

[4]Neutron Scattering Division, Oak Ridge National Laboratory, Oak Ridge, Tennessee 37831, USA

Corresponding Authors

*E-mail: spaddison@utk.edu; **E-mail: sokolov@utk.edu

Supplementary Methods

Conductivity Measurements Section

Spectra of complex conductivity measurements were probed using three setups to cover a wide frequency range from 0.1 Hz up to 30 GHz. An Alpha-A analyzer from Novocontrol with WinDETA Software from Novocontrol were utilized in the frequency range of 0.1 Hz to 10^6 Hz. The dielectric cell consisted of two electrodes with the fixed electrode distance of 0.4 mm and diameter of 10.2 mm were used. The samples were measured using a voltage with amplitude of 0.1 V. The standard calibration procedure was used before measurements. An Agilent RF Impedance Material Analyzer, E4991A with WinDETA Software from Novocontrol were used in the frequency range 10^6 Hz to $3{\cdot}10^9$ Hz. The cell was constructed from two APC-7 connectors. For the upper electrode, the inner pin of one connector was replaced by solid pin with diameter 3mm and space between inner pin and outer part was filled by Teflon. Regarding lower electrode, the pin in the other connector was removed and in the central space a metal cylinder was inserted, which can be moved changing the distance between the electrodes. As a result, the coaxial line was terminated by the plate capacitor with adjustable distance between the electrodes. The coaxial line was calibrated using standard procedure (Open/Short/50 Ohm) to move the reference plane

up to cell terminating the line. The cell was calibrated as well (Open and Short) for better precision. The samples were loaded between electrodes separated by 0.1 mm and measured with a voltage amplitude of 0.1 V. The Panoramic Network Analyzer, Agilent Technologies, E8364C with 85070E Dielectric Probe Kit were utilized for frequency measurements from $5 \cdot 10^8$ Hz up to $3 \cdot 10^{10}$ Hz. The Slim Probe with the Agilent Electronic Calibration module (ECal) were used for measurements of real and imaginary part of dielectric permittivity. The calibration was performed in standard procedure using Open, Short (Performance Probe Kit) and Load (Distilled water at 20^0C). Before each measurements the calibration was refreshed using ECal module. A Quattro temperature controller (Novocontrol) was used for temperature stabilization for the measurements from 10^{-1} Hz to 10^9 Hz. The samples were stabilized for 20 minutes at each temperature to reach precision ±0.2 K. The Presto, Julabo, W80 was used for temperature stabilization for the measurements from $5 \cdot 10^8$ Hz up to $3 \cdot 10^{10}$ Hz. The samples were stabilized for 40 minutes at each temperature to reach precision ±0.2 K. The conductivity spectra for pure PA are presented in the Figure 1 and for PA 85wt% in the Figure S1

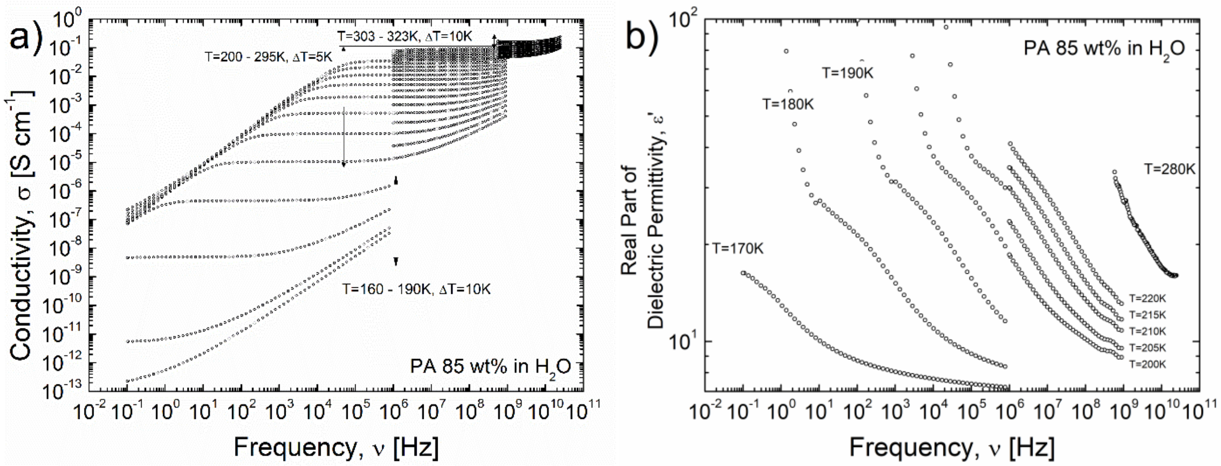

Supplementary Figure S1. Conductivity (a) and real part of the dielectric permittivity (b) spectra of PA85.

Quasielastic Neutron Scattering (QENS) Section

The QENS spectra (Fig.3 and S2) clearly show that $\chi''(Q,E) \sim E^1$ in the low energy range, and $\chi''(Q,E) \sim E^{-\beta}$ in the high energy. This behavior is typical for structural relaxation in liquids and is well described by the Cole-Davidson function[1]. Thus, for quantitative analysis of the QENS data

the intensity spectra at each Q value were fitted using Dave software[2] the following expression as a function of energy transfer, E

$$I(Q,E) = \left[x(Q)\delta(Q,E) + \left(1 - x(Q)\right)S(Q,E)\right] \otimes R(Q,E) + C(Q) \qquad \text{(S1)}$$

where $x(Q)$ is the fraction of the elastic scattering in the spectrum, $\delta(Q, E)$ is a delta-function describing the elastic peak at zero energy transfer, $S(Q, E)$ is a model scattering function, $C(Q)$ present a background offset. The signal was convoluted with resolution function $R(Q, E)$ derived from the data measured at T=20K. Considering the Cole-Davidson behavior of the susceptibility data and the normalization condition for the $S(Q, E)$, the following expression was used for quantitative analysis

$$S(Q,E) = \frac{-\mathrm{Im}[\chi_{\text{Cole-Davidson}}(Q,E)]}{\pi E} = \frac{(cos\varphi)^{\beta}\sin(\beta\varphi)}{\pi E}, \quad \varphi = \arctan(E/E_0) \qquad \text{(S2)}$$

where β and E_0 are the fitted parameters corresponding to the spectral stretching and position of the peak in the susceptibility presentation respectively. Thus, for each QENS spectrum there are four fitting parameters: $x(Q), C(Q), E_0(Q)$ and $\beta(Q)$. The Q dependence of the elastic component $x(Q)$, background offset $C(Q)$ and parameter $E_0(Q)$ are shown in Figure S2. Fitting of the intensity, $I(Q,E)$, and analysis of the spectral shape in the susceptibility data format $\chi''(Q,E) \sim I(Q,E) \times E$ at intermediate Q=0.5-1.1Å^{-1}, where the relaxation peak is well resolved, reveals stretching parameter is Q-independent and equals to $\beta \approx$ 0.68, indicating significant distribution of the proton relaxation process. For the fits at larger Q, the parameter β was fixed to be β=0.68. Having β and E_0 parameters, the energy corresponding to the maximum of the relaxation susceptibility spectra $E^{\chi}_{max}(Q)$ can be obtained as $E^{\chi}_{max}(Q) = E_0(Q)\sin\left[\frac{\pi}{2(\beta+1)}\right] / \sin\left[\frac{\pi\beta}{2(\beta+1)}\right]$, which is plotted in Fig 6. The parameter $E^{\chi}_{max}(Q)$ is close to Half-Width at the Half-Maximum (HWHM) of intensity data, but it additionally considers the distribution of relaxation processes in the spectra. In case of single relaxation process, where β=1, $S(Q,E)$ transforms to classical Lorentzian, with $E^{\chi}_{max}(Q) = E_0(Q)$ equals to HWHM of intensity data.

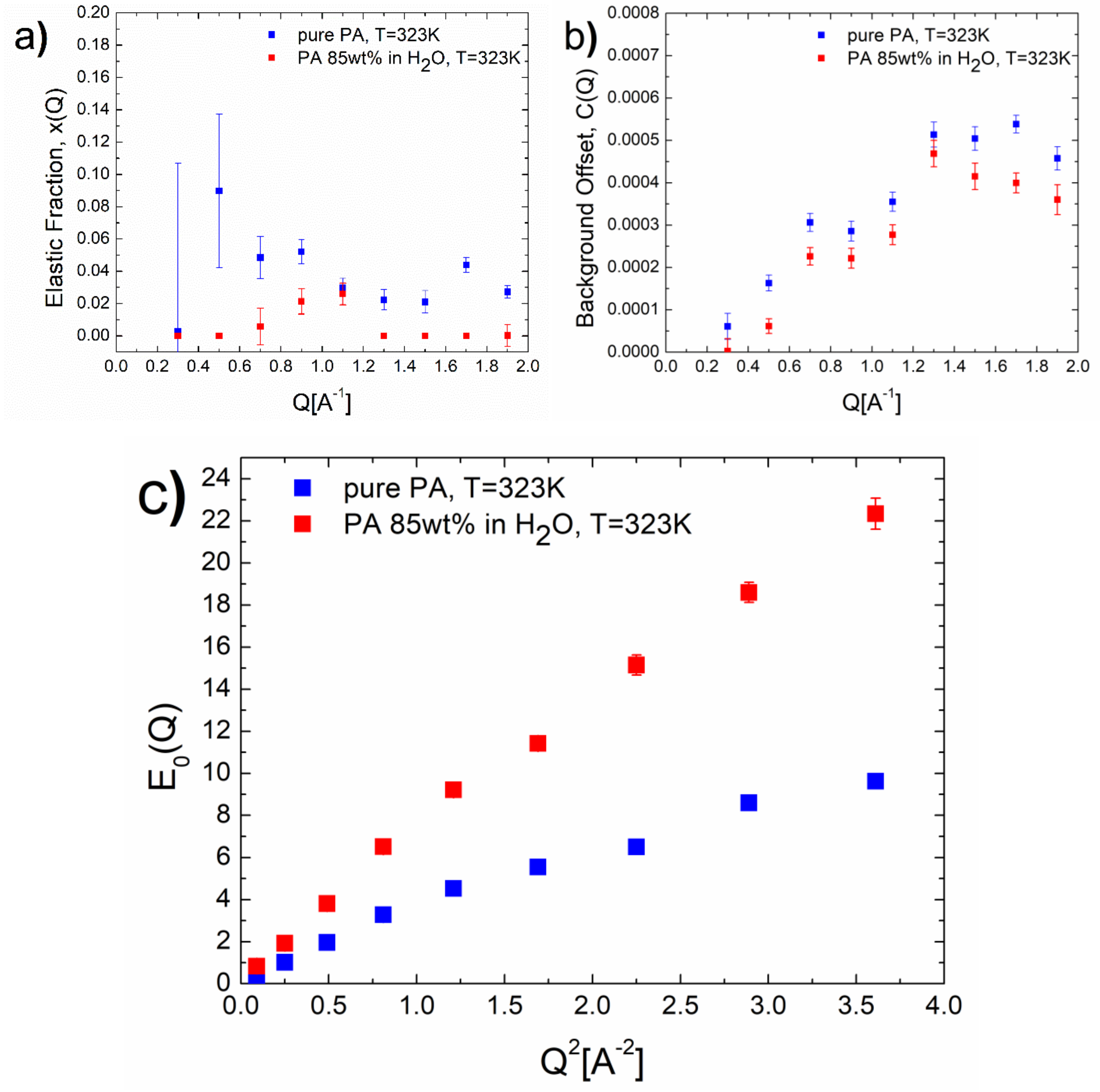

Supplementary Figure S2. The Q-dependence of the (a) fraction of the elastic scattering, $x(Q)$, (b) background offset C(Q) and (c) Cole-Davidson parameter $E_0(Q)$ for pure PA (blue symbols) and PA85 (red symbols). Error bars are standard deviation.

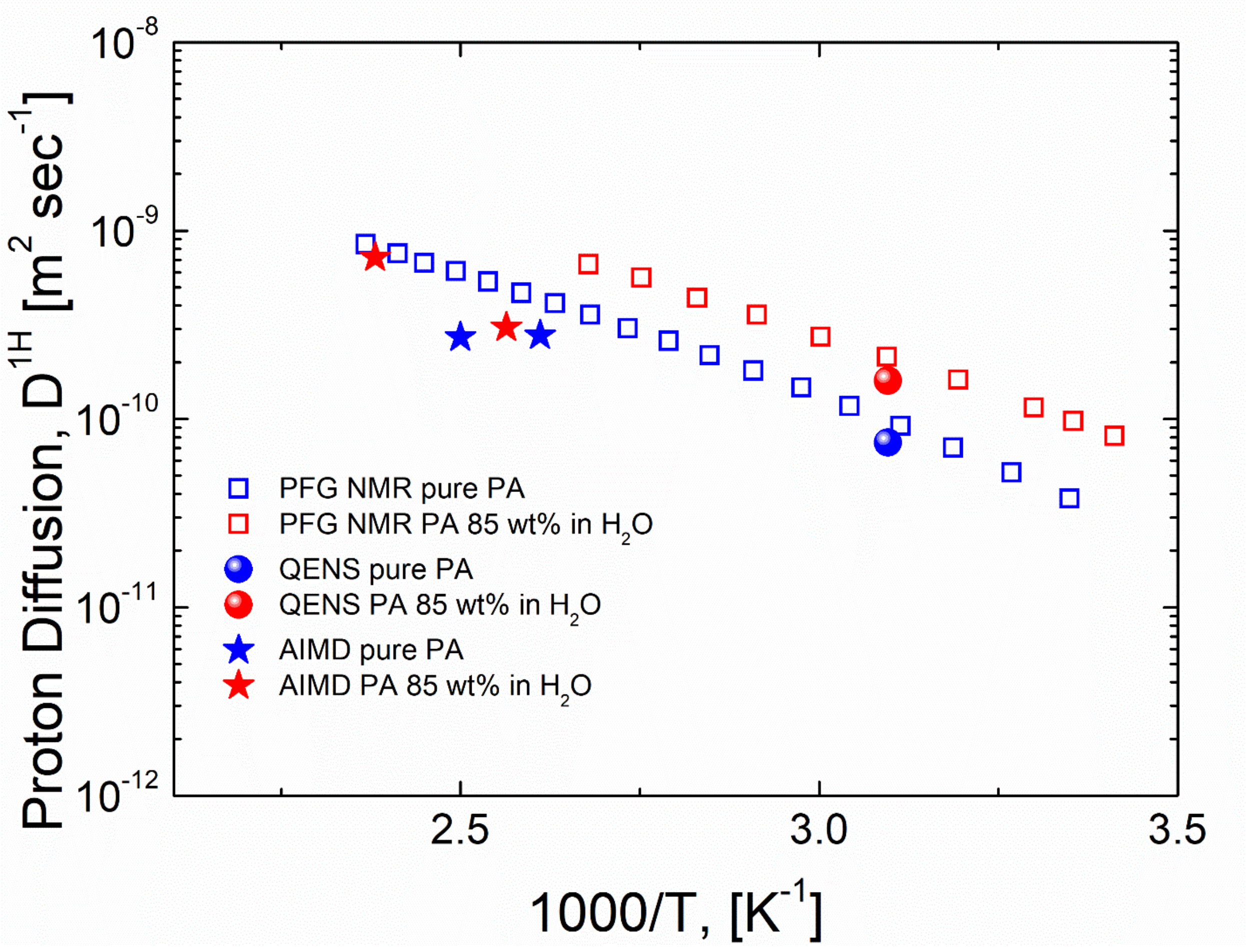

Supplementary Figure S3. Diffusivity data of proton ^1H for pure PA and PA85 obtained from PFG-NMR[3,4], QENS, and AIMD simulations.

***Ab Initio* Molecular Dynamic Simulations**

The self-part of the incoherent intermediate scattering function of protons can be computed by $ISF_s(Q,t) = \langle \exp(i\vec{\boldsymbol{Q}} \cdot [\vec{\boldsymbol{r}_i}(t) - \vec{\boldsymbol{r}_i}(0)]) \rangle$, in which i and $\vec{\boldsymbol{Q}}$ are the imaginary unit and the reciprocal lattice vector, respectively, $\vec{\boldsymbol{r}_i}$ is the coordinates of the ith proton. To illustrate the major processes in the proton motion, we first fitted the $ISF_s(Q,t)$ curves with two exponential terms and one stretched exponential term (i.e., $ISF_s(Q,t) = a_1 \exp(-t/\tau_1) + a_2 \exp(-t/\tau_2) + a_3 \exp(-[t/\tau_3]^{\beta})$) for various Q values as shown in Fig. S4a. The fitting results are shown in Table S1. The fastest process occurs within the first 100 fs, which corresponds to the covalent bond vibration. This process can be further verified by the vibrational density of state (VDOS) for protons in pure PA system at 400 K (see Fig. S4b), in which the relaxation time of the OD bond,

τ_1, is in good agreement with the fastest mode obtained from the fitting of $ISF_s(Q,t)$. Note that this process only accounts for a very small proportion of the $ISF_s(Q,t)$, especially for small Q values. Thus, we skipped this Q-independent vibrational mode in the latter fitting procedure by dismissing the first 100 fs, which improved the fitting results for small Q values but had a minimal effect on the ones for large Q values based on the comparison between the E_3 values from Table S1 and Table S3. Besides the vibrational mode, $ISF_s(Q,t)$ bears a fast relaxation process and a slow relaxation process, corresponding to proton rattling and proton diffusion processes, respectively. To estimate the relaxation times, the $ISF_s(Q,t)$ curves were fit to a modified Kohlrausch-Williams-Watts function $ISF_s(Q,t) = a_2\exp(-t/\tau_2) + a_3\exp[-(t/\tau_3)^{\beta}]$. The fast process has an exponential form, while the slow diffusive process features a stretched exponential form. The characteristic slow process relaxation time is given by $\tau_R = \tau_3/\beta\,\Gamma(1/\beta)$, where Γ is the Gamma function. Subsequently, the energy maximum of the relaxation spectra for the diffusive process E_3 can be calculated with $E_{max}^{\chi} = \hbar/\tau_R$, where $\hbar$ is Planck's constant divided by 2π. The estimated fitting parameters are presented in the Tables S2-S5.

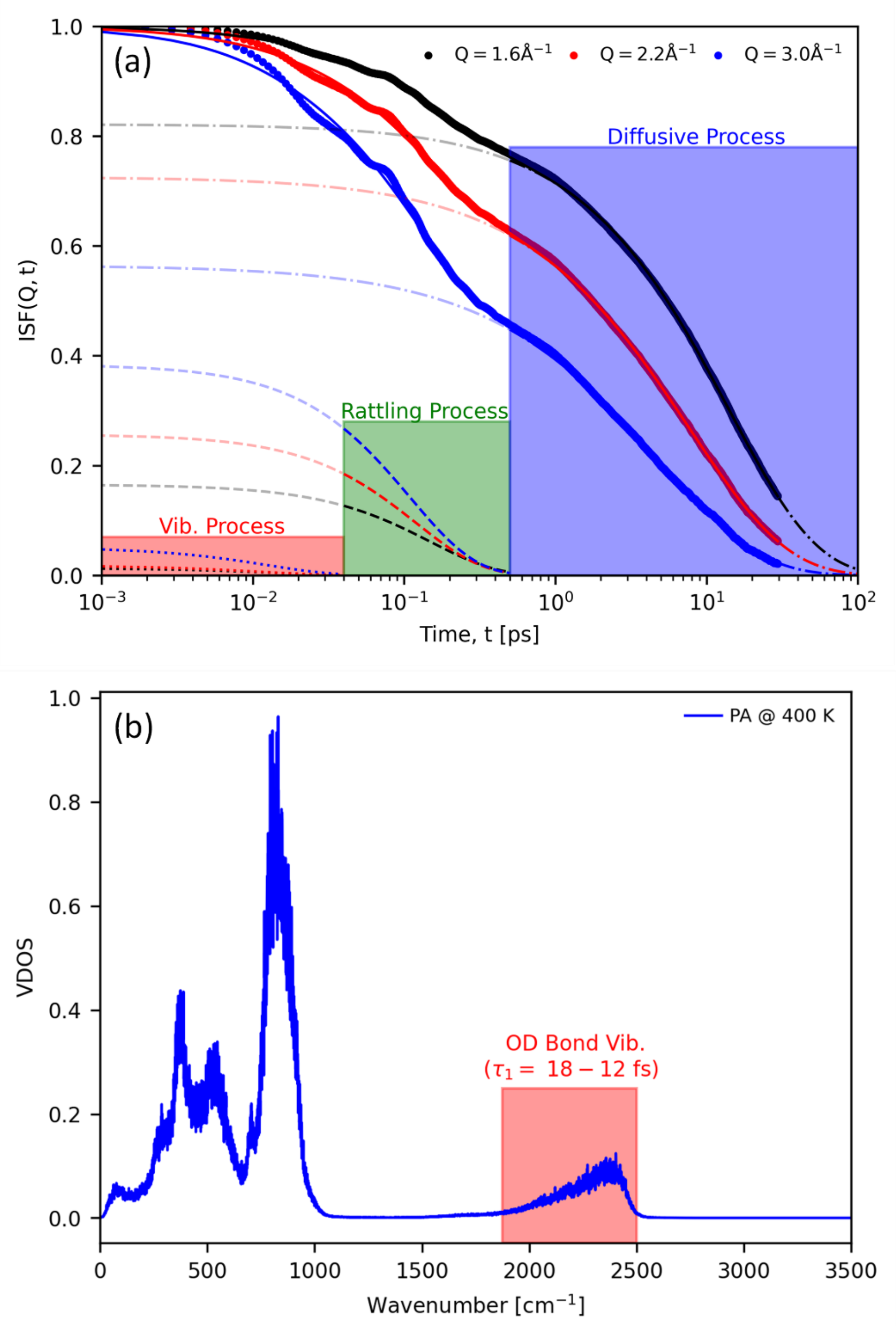

Supplementary Figure S4: (a) Illustration of fitting the self-intermediate scattering function $ISF_s(Q,t)$ of protons with $ISF_s = a_1 \exp(-t/\tau_1) + a_2 \exp(-t/\tau_2) + a_3 \exp(-[t/\tau_3]^{\beta})$ for three different Q values probed at 400 K for pure PA. (b) Vibrational density of state (VDOS) for protons in pure PA system at 400 K.

Supplementary Table S1: Relaxation times obtained by fitting the self-intermediate scattering function with $ISF_s = a_1\exp(-t/\tau_1) + a_2\exp(-t/\tau_2) + a_3\exp(-[t/\tau_3]^{\beta})$ for pure PA at 400 K.

Q[Å^{-1}]	a_1	τ_1[ps]	a_2	τ_2[ps]	a_3	τ_3[ps]	β	τ_R[ps]	E^{χ}_{max}	error
1.600	0.008	0.013	0.102	0.150	0.504	14.029	0.745	16.794	39.192	0.038
2.200	0.011	0.011	0.155	0.121	0.438	7.736	0.673	10.189	64.597	0.141
3.000	0.026	0.012	0.198	0.110	0.292	4.843	0.644	6.686	98.450	0.563

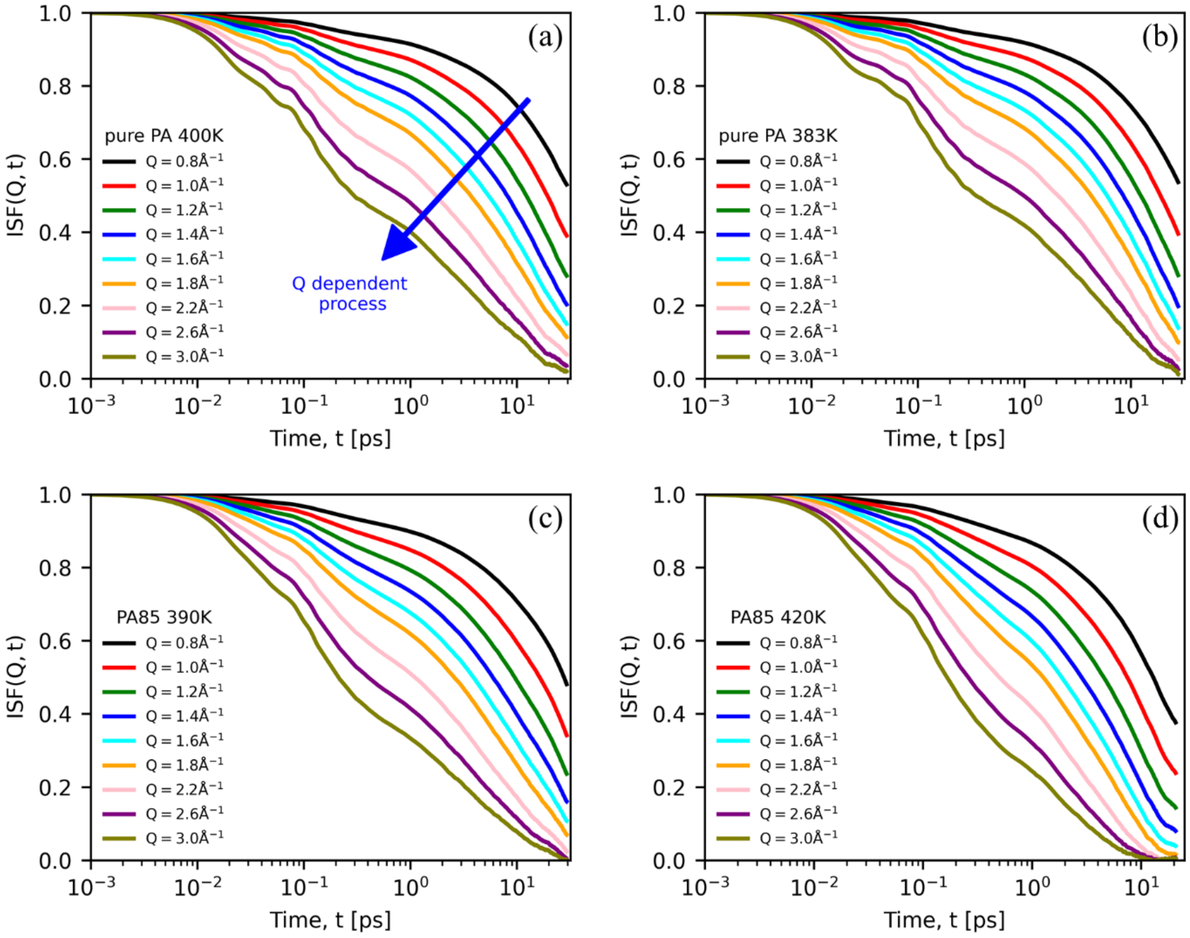

Supplementary Figure S5: Self-intermediate scattering function $ISF_s(Q,t)$ of the protons for different Q values probed at 400 K for pure PA (a), at 383 K for pure PA (b), at 390 K for aqueous PA85 (c), and at 420K for aqueous PA85.

Supplementary Table S2: Relaxation times obtained by fitting the self-intermediate scattering function with $ISF_s = a_2 \exp(-t/\tau_2) + a_3 \exp(-[t/\tau_3]^{\beta})$ for pure PA at 383 K.

Q[Å^{-1}]	a_2	τ_2[ps]	a_3	τ_3[ps]	β	τ_R[ps]	E^{χ}_{max}	error
0.800	0.042	0.169	0.787	54.923	0.827	60.834	10.820	0.005
1.000	0.069	0.166	0.827	34.383	0.824	38.175	17.242	0.007
1.200	0.099	0.162	0.818	24.066	0.816	26.890	24.478	0.017
1.400	0.127	0.155	0.769	18.146	0.803	20.510	32.093	0.039
1.600	0.157	0.147	0.735	14.380	0.784	16.529	39.823	0.070
1.800	0.182	0.138	0.684	11.785	0.764	13.829	47.595	0.106
2.000	0.206	0.131	0.638	9.898	0.744	11.868	55.462	0.141
2.200	0.227	0.124	0.582	8.480	0.727	10.364	63.510	0.164
2.400	0.252	0.119	0.541	7.392	0.716	9.167	71.802	0.206
2.600	0.278	0.114	0.501	6.534	0.707	8.191	80.355	0.260
2.800	0.304	0.110	0.464	5.835	0.701	7.381	89.178	0.372
3.000	0.322	0.105	0.421	5.250	0.695	6.695	98.308	0.470

Supplementary Table S3: Relaxation times obtained by fitting the self-intermediate scattering function with $ISF_s = a_2 \exp(-t/\tau_2) + a_3 \exp(-[t/\tau_3]^{\beta})$ for pure PA at 400 K.

Q [Å^{-1}]	a_2	τ_2[ps]	a_3	τ_3[ps]	β	τ_R[ps]	E^{χ}_{max}	error
0.800	0.028	0.194	0.439	56.930	0.841	62.341	10.558	0.011
1.000	0.042	0.178	0.444	35.936	0.818	40.097	16.415	0.011
1.200	0.064	0.164	0.483	24.769	0.795	28.197	23.344	0.023
1.400	0.081	0.150	0.470	18.181	0.770	21.198	31.050	0.045
1.600	0.099	0.138	0.462	13.975	0.742	16.780	39.226	0.075
1.800	0.120	0.128	0.462	11.131	0.715	13.818	47.636	0.125
2.000	0.138	0.121	0.440	9.136	0.690	11.724	56.144	0.170
2.200	0.156	0.115	0.416	7.706	0.671	10.174	64.693	0.251
2.400	0.180	0.110	0.401	6.662	0.658	8.984	73.262	0.359
2.600	0.201	0.106	0.374	5.876	0.649	8.042	81.849	0.507
2.800	0.221	0.103	0.347	5.257	0.643	7.273	90.497	0.810
3.000	0.242	0.099	0.322	4.745	0.638	6.626	99.333	0.910

Supplementary Table S4: Relaxation times obtained by fitting the self-intermediate scattering function with $ISF_s = a_2 \exp(-t/\tau_2) + a_3 \exp(-[t/\tau_3]^{\beta})$ for aqueous PA85 at 390 K.

Q[Å^{-1}]	a_2	τ_2[ps]	a_3	τ_3[ps]	β	τ_R[ps]	E^{χ}_{max}	error
0.800	0.063	0.274	0.692	49.644	0.864	53.477	12.308	0.024
1.000	0.084	0.227	0.672	31.759	0.812	35.597	18.491	0.042
1.200	0.108	0.192	0.673	21.622	0.767	25.288	26.029	0.096
1.400	0.134	0.169	0.665	15.467	0.734	18.763	35.080	0.177
1.600	0.163	0.154	0.642	11.616	0.713	14.448	45.556	0.280
1.800	0.190	0.145	0.590	9.158	0.704	11.526	57.105	0.412
2.000	0.223	0.139	0.546	7.552	0.704	9.511	69.204	0.594
2.200	0.255	0.133	0.497	6.459	0.708	8.090	81.361	0.850
2.400	0.284	0.127	0.448	5.661	0.712	7.053	93.327	1.157
2.600	0.310	0.121	0.404	5.036	0.713	6.262	105.116	1.648
2.800	0.330	0.115	0.360	4.524	0.712	5.633	116.840	1.716
3.000	0.348	0.110	0.323	4.087	0.708	5.116	128.662	2.152

Supplementary Table S5: Relaxation times obtained by fitting the self-intermediate scattering function with $ISF_s = a_2 \exp(-t/\tau_2) + a_3 \exp(-[t/\tau_3]^{\beta})$ for aqueous PA85 at 420 K.

Q[Å^{-1}]	a_2	τ_2[ps]	a_3	τ_3[ps]	β	τ_R[ps]	E^{χ}_{max}	error
0.800	0.048	0.197	0.623	22.769	0.847	24.833	26.505	0.028
1.000	0.074	0.178	0.654	14.061	0.825	15.595	42.207	0.113
1.200	0.100	0.168	0.615	9.496	0.819	10.588	62.164	0.300
1.400	0.132	0.166	0.554	6.982	0.839	7.659	85.940	0.473
1.600	0.178	0.169	0.506	5.570	0.886	5.914	111.306	0.585
1.800	0.222	0.167	0.452	4.680	0.934	4.830	136.271	0.774
2.000	0.255	0.157	0.409	3.977	0.949	4.072	161.648	1.161
2.200	0.277	0.143	0.376	3.370	0.929	3.487	188.760	1.500
2.400	0.294	0.130	0.347	2.870	0.896	3.027	217.450	1.694
2.600	0.311	0.121	0.319	2.482	0.868	2.666	246.851	2.112
2.800	0.328	0.113	0.290	2.190	0.850	2.383	276.157	2.817
3.000	0.345	0.107	0.262	1.966	0.839	2.156	305.308	3.435

Supplementary Table S6: Relaxation times obtained by fitting the Q-dependent $\mathrm{E}_{max}(\mathrm{Q}) = \hbar/\tau_R$ with

$E_{max}^{\chi}(Q) = \frac{\hbar D Q^2}{(1+D\tau Q^2)}$ for pure PA and aqueous PA85 at different temperatures.

System	T[K]	$1000/T$	D [$\times 10^{-10} m^2 s^{-1}$]	Error D	λ [Å]	Error λ	τ [ps]	Error τ
PA	383	2.611	2.78	0.0044	0.68	0.016	2.79	0.177
PA	400	2.500	2.72	0.0016	0.66	0.02	2.66	0.064
PA85	390	2.564	3.06	0.039	0.51	0.015	1.440	0.100
PA85	420	2.381	7.20	0.079	0.51	0.012	0.61	0.364

To verify the various relaxation time scales of proton motion, proton transfer population correlation functions (PCFs) were calculated from the trajectory. The PCF approach was proven capable of providing the characteristic time scales for proton transfer kinetics including proton rattling and long-range proton transport, which is defined as

$$C_c(t) = \frac{\langle h(0) \cdot H(t) \rangle}{\langle h \rangle} \tag{S3}$$

where $h(0) = 1$ if a given oxygen atom is protonated and 0 otherwise, $H(t) = 1$ if the protonated oxygen atom keeps its identity up to time *t*. The decay of $C_c(t)$ can be fitted to the tri-exponential form:

$$C_c(t) = \sum_{i=1}^{3} a_i \exp\left(-\frac{t}{\tau_i}\right) \tag{S4}$$

where the time constants τ_i correspond to the different time-scale processes. The results for pure PA at 400 K are shown in Figure S6a. The relaxation times are 0.32 ps, 2.20 ps, and 11.02 ps. The first fastest relaxation time is often assigned to the proton rattling process, which lies in the sub-picosecond regime. The slower one represents the hydrogen bond reorganization process and the slowest one comes from the long-range proton transport process, which is further confirmed by the similar characteristic relaxation time τ_{QENS} of 10.8 ps (Fig. 6). It is important to note that the proton transfer PCFs cannot detect vibrational motion because the proton transfer is the elementary step in this correlation function that has a larger time scale than vibrational motion. However, it is able to demonstrate additional details about proton transfers. Specifically, the hydrogen bond reorganization process can be easily detected via the proton transfer PCFs but is difficult to

distinguish from $ISF_s(Q,t)$ especially for small Q values. Interestingly, the fit of the proton transfer PCF that excludes proton rattling shows a single characteristic time (22.32 ps), meaning that there is only one slow process associated with this time scale.

The distribution of displacements of proton can be quantified by computing the self-part of the van Hove function defined as

$$G_s(r,t) = \frac{1}{N}\sum_{i=1}^{N}\langle\delta(r - |\vec{r_i}(t) - \vec{r_i}(0)|)\rangle \quad \text{(S5)}$$

where $4\pi r^2 G_s(r,t)$ is the probability that a particle has moved a distance *r* within the time *t*. The integral of $4\pi r^2 G_s(r,t)$ over all times must be unity, that is

$$\int_0^\infty 4\pi r^2 dr G_s(r,t) = 1 \quad \text{(S6)}$$

To estimate the length of proton transport at various characteristic times, the self-part van Hove functions at three characteristic time scales were determined as shown in Figure S6b. The van Hove functions demonstrate Gaussian distribution at short and long times, which correspond to local proton hopping and long-range proton diffusive motion. It is noted that there is a flat peak for the van Hove function at the intermediate time, which can probably be ascribed to the reorientation of covalent OH bond facilitating the escape of the “cage” for the proton. Importantly, the position of the peak for the shortest time scale is ~0.7 Å, which is in good agreement with the proton jump length. Indeed, this result illustrates that the short proton jump length is definitely the direct proton transfer.

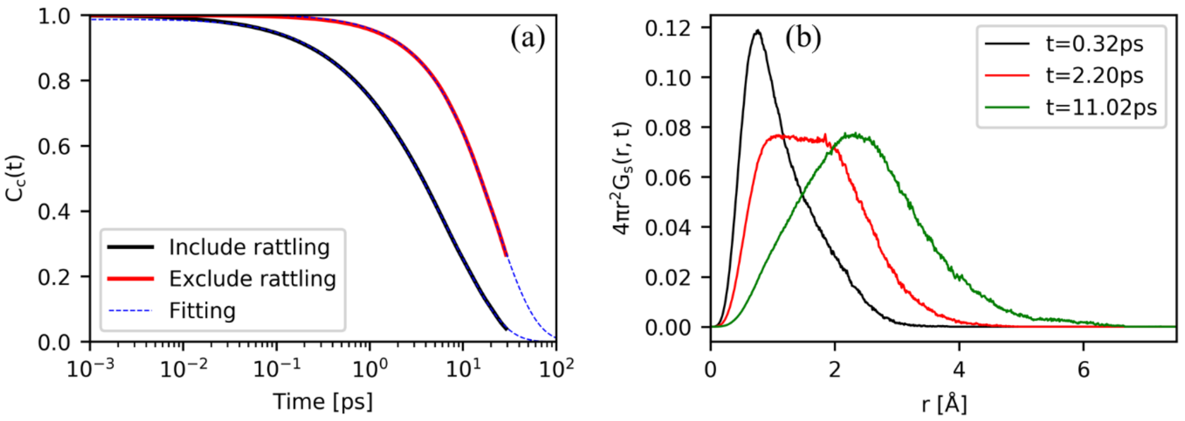

Supplementary Figure S6: (a) Proton transfer population correlation functions (both including and excluding proton rattling) and their triexponential fits for pure PA at 400 K. (b) The self-part of the van Hove function of the protons with three characteristic times obtained from triexponential fits of the population correlation functions in pure PA at 400 K.

In view of the correlation functions of the displacement vectors of protons, one may wonder if the negative distinct correlation of protons arises from the rotation of PA molecules. We calculated the distinct correlation function of the proton displacement vectors from the same PA molecule as shown in blue dash-dotted line (Fig. S7). The distinct diffusivity of protons of the same PA molecule appears to be a very small with a positive value, suggesting that the translational motion of the PA molecules may contribute more to the distinct proton diffusivity than the rotation of the PA molecules, and both are much slower than the proton self-diffusivity. Thus, it does not affect our conclusion whether we include all protons or exclude protons from the same PA molecule (Fig. S7). The former, however, is based on the standard definition of the correlation function and provides direct comparison to QNES data where all protons are equally included.

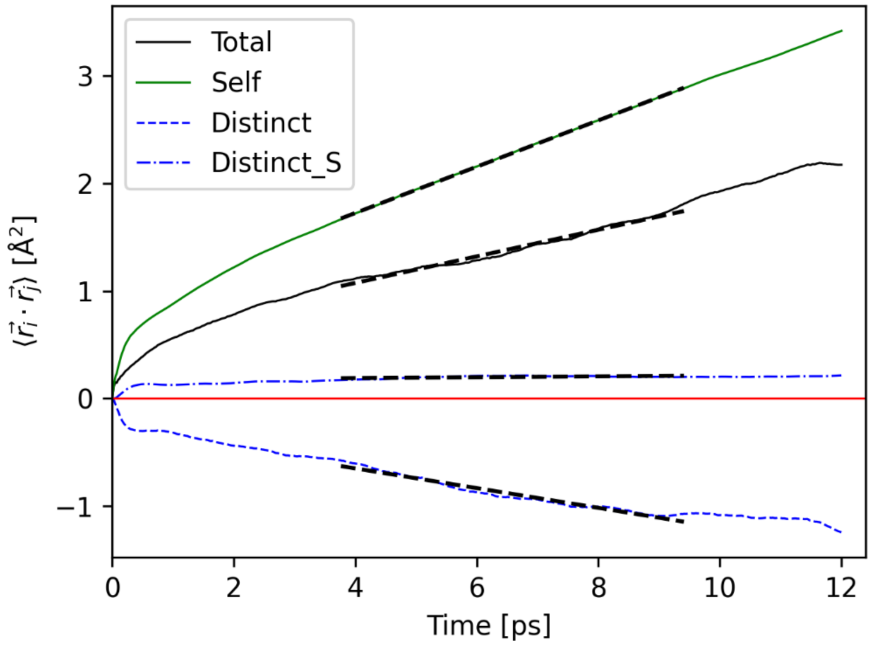

Supplementary Figure S7: Correlation functions of the proton displacement vectors excluding the protons from the same PA molecule. We obtained the inverse Haven ratio as $H^{-1} = 0.55$, which is slightly smaller than that computed by including all protons ($H^{-1} = 0.58$).

Supplementary References

1 Böttcher, C. J. F.; Bordewijk, P., *Theory of electric polarization. Volume II. Dilectric in time-dependent fields*. Elsevier Science Limited: Amsterdam, Vol. 2, p 561 (1978).

2 Azuah, R. T. et al. DAVE: A Comprehensive Software Suite for the Reduction, Visualization, and Analysis of Low Energy Neutron Spectroscopic Data. *J Res Natl Inst Stand Technol* **114**, 341-358, (2009)

3 Aihara, Y., Sonai, A., Hattori, M. & Hayamizu, K. Ion Conduction Mechanisms and Thermal Properties of Hydrated and Anhydrous Phosphoric Acids Studied With1H, 2H, And31P NMR. *J. Phys. Chem. B* **110**, 24999–25006, (2006).

4 Dippel, T., Kreuer, K. D., Lassègues, J. C. & Rodriguez, D. Proton Conductivity in Fused Phosphoric Acid; A 1H/31P PFG-NMR and QNS Study. *Solid State Ionics* **61**, 41-46, (1993).
